# Association between serum cytokine and neuron-specific enolase levels and the core symptoms of autism spectrum disorder

**DOI:** 10.1016/j.ibneur.2026.04.007

**Published:** 2026-04-21

**Authors:** Junhong Jiang, Li Yang, Baotian Wang, Ran Hua, De Wu

**Affiliations:** Department of Pediatrics, The first Affiliated Hospital of Anhui Medical University, Hefei, Anhui, PR China

**Keywords:** Serum cytokine, Neuron-specific enolase, Autism spectrum disorder

## Abstract

**Background:**

Investigating alterations in blood cytokine and brain enzyme levels in autism spectrum disorder (ASD) is crucial for understanding its complex pathogenesis.

**Method:**

Between January 2021 and December 2024, all children diagnosed with ASD at the First Affiliated Hospital of Anhui Medical University who met the inclusion criteria were consecutively enrolled. Among them, 26 presented with typical features and 15 with atypical features. A control group of 24 age- and sex-matched healthy children was recruited from unrelated families. Serum levels of cytokines (IL-2R, IL-1β, IL-6, IL-8, IL-10, TNF-α) and neuron-specific enolase (NSE) were measured by Enzyme-Linked Immunosorbent Assay, and their associations with the core symptoms of ASD were analyzed.

**Results:**

The ASD and control group showed statistically significant differences in serum IL-2R (*P* = 0.0097) and NSE (*P* < 0.0001) levels. In subgroup analyses, the typical and atypical ASD groups differed significantly in serum IL-2R levels (*P* = 0.0003), whereas the difference in NSE between subgroups was not significant after correction for multiple comparisons (*P* = 0.0437, *q* = 0.0874). Higher serum IL-2R levels were associated with more severe symptoms in children with ASD (CARS (*P* = 0.000), ABC (*P* = 0.000)). The correlation between TNF-α and ABC was of borderline significance (*P* = 0.030, *q* = 0.050). Higher serum NSE levels were associated with more severe symptoms (CARS (*P* = 0.048)). Serum NSE was a risk factor for ASD (*P* = 0.017). Serum NSE (*P* < 0.001), IL-2R (*P* = 0.014), and TNF-α (*P* < 0.001) had significant diagnostic value for ASD. The combined measurement of serum NSE + IL-2R + TNF-α (*P* < 0.001), NSE + IL-2R (*P* < 0.001), NSE + TNF-α (*P* < 0.001), and IL-2R + TNF-α (*P* < 0.001) also showed significant diagnostic value.

**Conclusion:**

In this cross-sectional study, serum cytokines (IL-2R, TNF-α) and NSE were found to be associated with ASD. The combination of NSE with cytokines such as IL-2R and TNF-α may may be associated with distinct clinical features in children with ASD.

## Highlights

• Serum IL-2R and NSE levels were significantly elevated in children with ASD compared to controls, and differed between typical and atypical ASD subgroups.
• Elevated serum levels of IL-2R, TNF-α, and NSE were positively correlated with the severity of core ASD symptoms as measured by standardized behavioral scales.
• Serum NSE, IL-2R, and TNF-α, both individually and in combination, showed significant diagnostic value for autism spectrum disorder.

## 1 Introduction

Autism Spectrum Disorder (ASD) is a group of neurodevelopmental conditions characterized by marked heterogeneity in clinical presentation and severity. Its core features encompass persistent deficits in social communication and interaction, alongside restricted, repetitive patterns of behavior and interests (American Psychiatric Association, 2022, World Health Organization, 2018). With a global prevalence of approximately 1%, ASD poses a significant public health challenge (Zeidan et al., 2022). While current diagnostic systems (DSM-V-TR) define ASD as a single diagnostic entity covering a broad spectrum of patients, considerable phenotypic heterogeneity persists in clinical practice. Some patients exhibit severe and classic symptoms, while others present with milder or atypical features (Masi et al., 2017a). To explore the biological underpinnings of this heterogeneity, we employed the ICD-10 criteria to classify patients into typical and atypical ASD subgroups. This classification system was chosen because it provides clear operational definitions (e.g., symptom profiles) that allow for meaningful subgroup comparisons.

The etiology of ASD remains incompletely understood, with evidence pointing to a complex interplay of genetic, neurological, and environmental factors (Reynoso et al., 2017). Twin studies, which show higher concordance in monozygotic than dizygotic twins, underscore a substantial genetic contribution; however, the lack of absolute concordance highlights the important role of non-genetic influences (Bölte et al., 2019). Among these, prenatal exposures (e.g., to valproic acid) and immune dysregulation, involving either the maternal or the affected individual (EmbertiGialloreti et al., 2019).

In recent years, immune system dysfunction has gained increasing attention in ASD pathogenesis (Hughes et al., 2023). Inflammation, a critical host defense mechanism, involves tightly regulated innate and adaptive immune responses. Dysregulation of these pathways can adversely impact early brain development and neural function (Masi et al., 2017b, Onore et al., 2012). Proposed mechanisms are multi-level, spanning systemic to cellular scales, and operate during key developmental windows from fetal to postnatal life (Goines and Van de Water, 2010). Cytokines, as pivotal immune signaling molecules, play a central role. They can be categorized into groups such as those linked to adaptive immunity (e.g., IL-2, IL-2R), pro-inflammatory cytokines (e.g., IL-1β, IL-6, IL-8, TNF-α), and anti-inflammatory cytokines (e.g., IL-10) (Turner et al., 2014). Cytokines are known to regulate neural development, synaptic plasticity, and higher-order brain functions related to cognition and emotion (Manzardo et al., 2012), potentially underpinning specific behavioral phenotypes in ASD, including emotional and sleep dysregulation (Baker et al., 2013). Consistent with this, numerous studies report significant alterations in cytokine profiles in individuals with ASD across both peripheral (e.g., serum, plasma) and central nervous system compartments (Wei et al., 2012, Vargas et al., 2005, Sreenivas et al., 2024).

Concurrently, as a biomarker of neuronal damage, neuron-specific enolase (NSE) has been associated with cognitive deficits in various neurological conditions (Isgrò et al., 2015, Stancioiu et al., 2023). However, the interrelationship between immune dysfunction, neuronal damage (as suggested by elevated NSE), and the core symptoms of ASD remains unclear. It is plausible that immune dysregulation in ASD is not merely a comorbid feature but may also contribute to ongoing neuronal disturbances, thereby exacerbating the behavioral manifestations of the disorder.

To address this, the present study concurrently profiles a specific panel of peripheral immune markers (IL-2R, IL-1β, IL-6, IL-8, TNF-α, IL-10) and NSE in children with ASD. Our objectives are to investigate the correlations of these biomarkers with ASD diagnosis and core symptom severity, and to explore the interrelationship between immune dysfunction, neuronal damage (as suggested by elevated NSE), and the core symptoms of ASD.

## 2 Materials and methods

### 2.1 Study subjects

Between January 2021 and December 2024, a total of 258 children diagnosed with Autism Spectrum Disorder (ASD) at the First Affiliated Hospital of Anhui Medical University, yielding a final sample of 41 participants (26 with typical ASD and 15 with atypical ASD) who met the inclusion and exclusion criteria. A control group of 24 healthy children, matched for age and sex, were recruited from unrelated families. All participants were aged 1–6 years (Table 1.1), and informed consent was obtained from their parents. The core symptoms of ASD based on the DSM-V-TR, and classified children with typical and atypical ASD according to ICD-10 (American Psychiatric Association, 2022, World Health Organization, 2018, Grzadzinski et al., 2013).

**Table 1.1:** Demographic and Clinical Characteristics of the Study Subjects.

Clinical Characteristics	**Autism group**	**Control group**
**Cases**	41	24
**Sex**		
Male	31	15
Female	10	9
**Age (years)**		
＜2	3	1
2–3	27	7
＞3	11	16
**Etiology**		
Hereditary diseases	0	N
Non-genetic diseases	41	N
**Types**		
Typical	26	N
Atypical	15	N
**Complications**	0	N

Inclusion criteria: (a) A formal diagnosis of autism established by a qualified professional; (b) The ability to comprehend and use Chinese, required for both the child and their parents to ensure adequate understanding of study procedures.

Exclusion criteria: (a) Other neurological disorders and genetic metabolic syndrome diseases, (b) Other severe psychiatric disorders (such as depression, anxiety, attention deficit hyperactivity disorder, and bipolar disorder), (c) Personal or family history of autoimmune diseases, (d) History of severe allergies or atopic diseases (such as rhinitis, asthma, allergic enteritis, eczema), (e) Recent vaccination, (f) Recent use of medications affecting the immune system (such as anti-inflammatory drugs, immunostimulants).

### 2.2 Measurement of serum cytokines and neuron-specific enolase

Blood samples were collected from children with ASD (both typical and atypical) and healthy controls using serum separation tubes. The samples were centrifuged at 1000 × g for 20 min, and the resulting supernatant was collected. The serum levels of TNF-α, IL-1β, IL-2R, IL-6, IL-8, IL-10, and NSE were measured using commercial Enzyme-Linked Immunosorbent Assay (ELISA) kits (Siemens Healthcare Diagnostics Products Limited, Llanberis, UK), strictly following the manufacturer's protocols. The lower limits of detection for TNF-α, IL-1β, IL-2R, IL-6, IL-8, and IL-10 were respectively 4, 5, 5, 2, 5, and 5 pg/mL.

### 2.3 Standardized diagnosis and severity assessment of autism

The measured levels of serum cytokines (TNF-α, IL-1β, IL-2R, IL-6, IL-8, IL-10) and NSE were analyzed for their association with scores from the Childhood Autism Rating Scale (CARS) and the Autism Behavior Checklist (ABC). To ensure diagnostic consistency, all children with ASD (both typical and atypical) underwent an initial developmental clinical interview. This interview confirmed the core symptoms of ASD based on the DSM-V-TR, and classified children with typical and atypical ASD according to ICD-10. Furthermore, all autistic children were assessed using the following two instruments:

(a) Childhood Autism Rating Scale (CARS) (Scholper et al., 2010): This 15-item screening tool uses a 4-point scale for each item. Severity is classified as mild-to-moderate (scores of 30–36, with fewer than 5 items ≥ 3) or severe (scores ≥ 36, with at least 5 items > 3).

(b) Autism Behavior Checklist (ABC) (Abdelmageed et al., 2024): This 57-item checklist, used for secondary screening, also employs a 4-point scale per item. Total scores are interpreted as suspect (53−67) or indicative of a confirmed diagnosis (≥ 68).

### 2.4 Statistical analysis

Continuous data were summarized as mean ± standard deviation. All statistical analyses were conducted with SPSS 19, and graphs were created with GraphPad Prism 8.0.2. Normality of distribution was assessed using the Shapiro-Wilk test. Inter-group differences in the concentrations of serum cytokines (IL-2R, IL-8, TNF-α) and NSE were normally distributed (*P* > 0.05) and analyzed by unpaired *t*-tests. To evaluate the association between specific biomarkers (IL-2R, TNF-α, and NSE) and the risk of autism, Spearman's correlation and binary logistic regression analyses were employed. Results were considered significant if *P* < 0.05. Given the number of statistical tests performed across the study (ASD vs. control and typical vs. atypical ASD comparisons for seven biomarkers, and biomarker correlations with CARS and ABC scores), we applied the Benjamini–Hochberg false discovery rate (FDR) correction to control for multiplicity. Adjusted *q*-values were calculated for each family of tests; a *q* < 0.05 was considered statistically significant. Results are reported with both uncorrected *P* -values and FDR-adjusted *q*-values.

## 3 Results

### 3.1 Participant demographics and baseline data

The demographic characteristics of the Autism Spectrum Disorder (ASD) and control groups are presented in Table 1.1. In the ASD group, the male-to-female ratio was 31:10, consistent with the known male predominance in ASD (Zeidan et al., 2022), while the control group had a more balanced sex distribution (15:9). Age distributions showed some variation between groups, with a higher proportion of children aged 2–3 years in the ASD group and a higher proportion of children over 3 years in the control group.

### 3.2 Serum cytokine and neuron-specific enolase levels in autism spectrum disorder compared with control children

Table 1.2 details the serum levels of cytokines (TNF-α, IL-1β, IL-2R, IL-6, IL-8, IL-10) and NSE in both the ASD and control groups. Unpaired *t*-tests revealed that children with ASD had significantly higher serum levels of IL-2R (*P* = 0.0097, *q* = 0.0146) and NSE (*P* < 0.0001, *q* = 0.0003) compared to the control group. In contrast, no statistically significant difference was found in serum TNF-α levels between the two groups. Furthermore, the concentrations of IL-1β, IL-6, IL-10, and IL-8 in a substantial proportion of samples fell below the lower detection limit and were therefore excluded from statistical analysis. Specifically, for IL-1β, levels were below the detection limit in 26 of 41 ASD participants and in all 21 control participants. For IL-6, levels were below the limit in 22 ASD participants and in all controls. For IL-10, levels were below the limit in 37 ASD participants and in all controls. For IL-8, levels were below the limit in all controls. Given the high proportion of non-detectable values, particularly in the control group, these cytokines were not subjected to further statistical analysis.

**Table 1.2:** Serum cytokine and NSE levels between ASD and controls children.

**Group**	**NSE**	**IL-2R**	**TNF-α**
**ASD**	26.53 ± 10.29	734.51 ± 352.86	12.57 ± 4.76
**Control**	8.39 ± 4.63	527.13 ± 183.78	8.37 ± 2.69
***t***	8.146	2.669	1.602
***P***	＜0.0001	0.0097	0.1142
***q***	0.0003	0.0146	0.1142

### 3.3 Serum cytokine and neuron-specific enolase levels in typical compared with atypical autism

Table 1.3 details the serum levels of cytokines (TNF-α, IL-1β, IL-2R, IL-6, IL-8, IL-10) and NSE in the typical and atypical autism subgroups. Unpaired *t*-tests demonstrated that the typical autism group had significantly higher serum levels of IL-2R (*P* = 0.0003, *q* = 0.0012) compared to the atypical autism group. However, the difference in NSE between subgroups (*p* = 0.0437, *q* = 0.0874) did not survive correction and should be interpreted with caution (Table 1.3). No statistically significant differences were observed in the levels of IL-8 and TNF-α between the two subgroups. Additionally, a substantial proportion of samples had cytokine concentrations below the lower detection limit and were therefore excluded from statistical analysis. Specifically, IL-1β levels were below the detection limit in 17 out of 26 participants in the typical autism group and 9 out of 15 in the atypical autism group; IL-6 levels were below the limit in 19 typical and 3 atypical cases; and IL-10 levels were below the limit in 23 typical and 14 atypical cases.

**Table 1.3:** Serum cytokine and NSE levels between typical and atypical autism.

**Group**	**NSE**	**IL-2R**	**IL-8**	**TNF-α**
**Typical ASD**	28.98 ± 11.79	876.23 ± 368.28	26.01 ± 17.98	13.54 ± 4.64
**Atypical ASD**	22.29 ± 4.90	488.87 ± 107.63	16.66 ± 13.51	10.94 ± 4.64
***t***	2.085	3.958	1.746	1.080
***P***	0.0437	0.0003	0.0886	0.287
***q***	0.0874	0.0012	0.1181	0.287

### 3.4 Correlation analysis between serum cytokines/neuron-specific enolase and clinical symptoms in autism spectrum disorder

Table 1.4 details the correlation analyses between serum biomarkers (TNF-α, IL-2R, NSE) and clinical symptom scores in ASD. Spearman correlation analysis demonstrated a significant positive correlation between elevated serum IL-2R levels and the severity of both social impairment and stereotyped behaviors, as measured by the CARS (r = 0.588, *P* < 0.001, *q* < 0.001) and ABC (r = 0.575, *P* < 0.001, *q* < 0.001) scales. Similarly, increased NSE levels showed a positive correlation with clinical severity, as indicated by the CARS score (r = 0.311, *P* = 0.048, *q* = 0.048). However, the correlation between TNF-α and ABC (r = 0.340, *P* = 0.030, *q* = 0.050) did not meet the adjusted significance threshold.

**Table 1.4:** Correlation between CARS and ABC Scores and Serum Levels of Cytokines and NSE.

**Serum indicators**	**NSE**	**IL-2R**	**CARS**	**ABC**
**NSE**	r = 1	r = 0.315,*P* = 0.045, *q*= 0.056	r = 0.311,*P* = 0.048, *q*= 0.048	-
**IL-2R**	r = 0.315,*P* = 0.045,*q*= 0.056	r = 1	r = 0.588,*P* = 0.000, *q*= 0.000	r = 0.575,*P* = 0.000, *q*= 0.000
**TNF-α**	-	-	-	r = 0.340,*P* = 0.030, *q*= 0.050

To characterize the relationship between the two clinical severity measures, we compared CARS and ABC scores in the same children (Table 1.5). ABC scores were significantly higher than CARS scores (32.88 ± 7.90, *P* < 0.0001). The two scales showed a moderate positive correlation (Pearson r = 0.464, *P* = 0.0023; Spearman ρ = 0.480, *P* = 0.0015).

**Table 1.5:** Relationship between CARS and ABC scores in ASD children.

**Analysis**	**Result**	**95% CI**	*P*
**CARS score**	35.29 ± 4.08	-	-
**ABC score**	68.17 ± 7.35	-	-
**Paired difference (ABC-CARS)**	32.88 ± 7.90	(30.39, 35.37)	< 0.0001
**Pearson correlation (CARS vs. ABC)**	r = 0.464	(0.185, 0.678)	0.0023
**Spearman correlation (CARS vs. ABC)**	ρ = 0.480	-	0.0015

### 3.5 Multivariate analysis of autism spectrum disorder risk in relation to serum cytokines and neuron-specific enolase

Table 1.6 presents the results of the regression analysis examining the association between serum biomarkers (TNF-α, IL-2R, NSE) and the risk of ASD. Binary logistic regression identified serum NSE as an independent risk factor for ASD (*P* = 0.017). After adjusting for IL-2R and TNF-α, serum NSE was associated with a 2.137-fold increased risk of ASD (OR = 2.137, 95% CI: 1.146 – 3.985). In contrast, neither serum IL-2R nor TNF-α demonstrated independent predictive value for the disorder.

**Table 1.6:** Binary logistic regression analysis of ASD with serum cytokines and NSE.

**Serum indicators**	***B***	**S.E**	***P***	**OR**	**95% CI for OR** **Lower Upper**	
**NSE**	0.759	0.318	0.017	2.137	1.146	3.985
**IL-2R**	-0.001	0.003	0.773	0.999	0.993	1.005
**TNF-α**	0.404	0.208	0.052	1.498	0.997	2.251

**Fig. 1.1:**
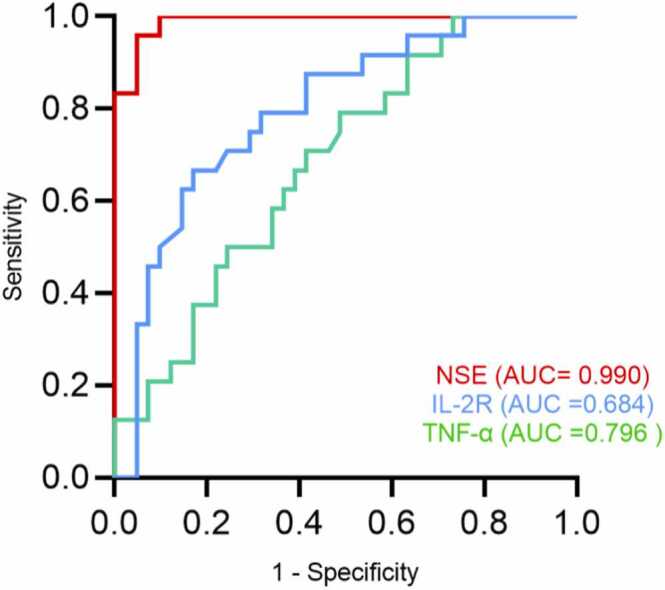
ROC curves of IL-2R, TNF-α and NSE measured separately.

We performed Receiver Operating Characteristic (ROC) curve analysis to assess the diagnostic utility of serum IL-2R, TNF-α, and NSE for ASD. As shown in Fig. 1.1, all three biomarkers individually showed significant discriminatory power: NSE (AUC = 0.990, 95% CI: 0.974–1.000, *P* < 0.001), TNF-α (AUC = 0.796, 95% CI: 0.685–0.908, *P* < 0.001), and IL-2R (AUC = 0.684, 95% CI: 0.556–0.813, *P* = 0.014). Their optimal cut-off values were determined with the following performance characteristics: NSE at 16.96 (Sensitivity: 95.1%, Specificity: 95.8%, Youden's index: 0.91); TNF-α at 8.165 (82.9%, 66.7%, 0.496); IL-2R at 648.5 (51.2%, 79.2%, 0.304).

**Fig. 1.2:**
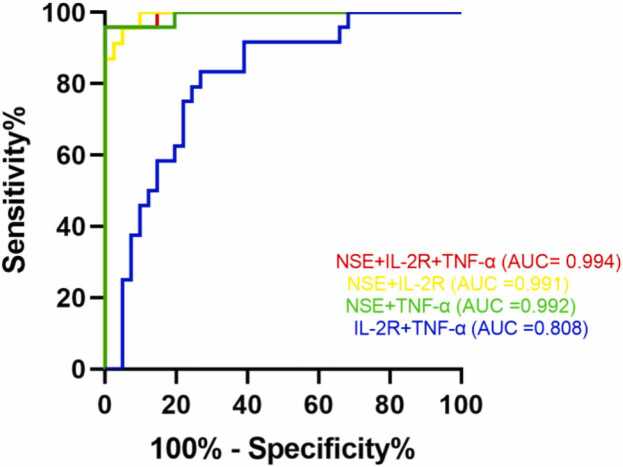
ROC curves for the combined measurement of IL-2R, TNF-α and NSE.

The combinations of these biomarkers were also evaluated (Fig. 1.2). The panel of all three (NSE + IL-2R + TNF-α) achieved the highest AUC of 0.994 (95% CI: 0.981–1.000, *P* < 0.001). The two-biomarker panels of NSE + TNF-α (AUC = 0.992) and NSE + IL-2R (AUC = 0.991) also showed near-perfect discrimination, while the IL-2R + TNF-α combination yielded an AUC of 0.808 (95% CI: 0.700–0.915, *P* < 0.001).

## 4 Discussion

This study found statistically significant differences in serum IL-2R levels between children with autism spectrum disorder (ASD) and healthy controls, as well as between typical and atypical autism subgroups. Furthermore, elevated serum IL-2R levels were positively correlated with the worsening of core symptoms. ROC curve analysis indicated that serum IL-2R holds considerable diagnostic value for ASD, with an optimal cut-off value of 648.5. Collectively, these results suggest an association between serum IL-2R levels and the core symptoms and severity of ASD. IL-2 is a pleiotropic cytokine that, upon binding to its receptor IL-2R, activates downstream signaling pathways (e.g., JAK-STAT) to regulate T-cell proliferation, differentiation, and immune tolerance. Within the central nervous system (CNS), IL-2 and its receptor are produced not only by immune cells but also by neurons, astrocytes, and microglia. Our findings imply that the serum IL-2/IL-2R system may be involved in immune dysregulation associated with ASD. A substantial body of literature identifies IL-2 as a key messenger bridging the immune and nervous systems. Its effects on the CNS are highly context-dependent: in the healthy brain, it acts as an endogenous regulator maintaining delicate balance, whereas under pathological conditions, its dysregulation can become a critical driver in the vicious cycle of neuroinflammation and neurodegeneration (Ellery and Nicholls, 2002). Clinical evidence shows that high-dose intravenous IL-2 administration for certain cancers can induce severe neuropsychiatric side effects, including cognitive deficits, affective symptoms, and sleep disturbances (Denicoff et al., 1987). Additionally, some studies have reported significantly elevated IL-2 levels in the hippocampal regions of patients with Alzheimer's disease compared to controls, suggesting marked immune system activation (Araujo and Lapchak, 1994). Singh et al. were the first to report significantly higher serum IL-2 concentrations in autistic children versus controls, but found no difference in IL-2R levels (Singh et al., 1991). However, some studies have reported no significant differences in serum IL-2 levels between individuals with autism and control groups (Ashwood et al., 2010, Li et al., 2009). Additionally, a meta-analysis further indicated no significant alterations in peripheral blood levels of either IL-2 or IL-2R in ASD patients (Saghazadeh et al., 2019). In this context, our finding of a positive correlation between serum IL-2R levels and core symptom severity suggests that the IL-2/IL-2R axis may still contribute to immune dysregulation associated with symptom expression, at least in a subset of individuals. Rather than serving as a standalone diagnostic discriminator, IL-2R may hold greater utility in characterizing clinical heterogeneity or severity. Future studies with larger, well-phenotyped cohorts are needed to explore whether combining IL-2R with other cytokines could help refine ASD subtype classification, and to elucidate the mechanistic role of IL-2/IL-2R signaling within specific cell types and neural circuits through approaches such as single-cell sequencing and functional experiments.

Consistent with previous studies reporting positive associations between elevated serum TNF-α levels and ASD symptom severity (Xie et al., 2017, Olmos and Lladó, 2014, Kim et al., 2009), our results indicate that higher serum TNF-α levels are associated with more severe core symptoms (TNF-α -ABC: *P* = 0.030) in children with ASD. However, this correlation did not meet the adjusted significance threshold after FDR correction (*q* = 0.050). ROC curve analysis suggested diagnostic relevance, with an optimal cut-off value of 8.165. These observations align with the hypothesis that TNF-α may contribute to neuropathology through disruption of synaptic plasticity and glutamate-mediated excitotoxicity (Xie et al., 2017, Olmos and Lladó, 2014, Kim et al., 2009). Nevertheless, several findings warrant cautious interpretation. No significant differences in serum TNF-α levels were observed between ASD and control groups, nor between typical and atypical autism subgroups. The observation that a biomarker correlates with symptom severity despite showing no group-level difference is not inherently contradictory. A biomarker may reflect disease severity or specific phenotypic features without serving as a reliable diagnostic differentiator between clinical and non-clinical populations. Our findings are consistent with those of Singh et al., who similarly reported no significant difference in plasma TNF-α levels between autistic individuals and controls (Ashwood et al., 2010). In contrast, Jyonouchi et al. documented elevated serum TNF-α levels in children with ASD compared to both healthy siblings and unrelated controls (Jyonouchi et al., 2001). These discrepancies may reflect variability in sample characteristics, age ranges, inflammatory endophenotypes, or the possibility that TNF-α elevations are present only in a subset of individuals with ASD. Notably, Chez et al. detected elevated TNF-α in the cerebrospinal fluid (CSF) of autistic children (Chez et al., 2007), suggesting that compartmentalized fluid measures may more directly capture localized neuroinflammatory processes, whereas peripheral blood levels are more susceptible to systemic influences. Taken together, these results suggest that TNF-α may serve as a marker of symptom severity in ASD rather than a robust case-control discriminator. Future studies should focus on well-defined clinical subgroups and consider integrating peripheral and central inflammatory measures to clarify the contexts in which TNF-α has utility as a biomarker.

This study found statistically significant differences in serum neuron-specific enolase (NSE) levels between children with ASD and the control group, as well as between the typical and atypical autism subgroups (*P* = 0.0437). However, the difference in NSE between the typical and atypical autism subgroups did not survive correction (*q* = 0.0874) and should be interpreted with caution. Higher serum NSE levels were correlated with more severe social impairment and repetitive stereotyped behaviors in children with ASD. Furthermore, serum NSE was identified as a risk factor for ASD, increasing the likelihood of the disorder. ROC curve analysis indicated that serum NSE has considerable diagnostic value for ASD, with an optimal cut-off value of 16.96. These results suggest a positive correlation between serum NSE levels and the core symptoms and severity of ASD, supporting the potential utility of serum NSE as an auxiliary biomarker for diagnosis and clinical characterization in ASD, though validation in larger independent cohorts is needed. Previous studies have reported elevated NSE levels in the serum or cerebrospinal fluid of children with ASD, which may reflect neuronal damage or impaired blood-brain barrier integrity (Onore et al., 2012). In other neurodevelopmental disorders, such as cerebral palsy and epilepsy, elevated NSE is also recognized as a marker of neuronal stress (Masi et al., 2017b). As a biomarker of neuronal damage or aberrant activity, increased NSE may indicate underlying neuroinflammatory processes, oxidative stress, or synaptic dysfunction in autism (Masi et al., 2017b, Onore et al., 2012). The present findings further support the hypothesis that neuronal injury or synaptic dysfunction is directly involved in the core symptomatology of ASD. Future longitudinal studies are warranted to examine whether dynamic changes in NSE levels may serve as a marker of treatment response, and to explore potential interventions that may influence NSE production or clearance.

We compared ABC and CARS scores in the same children using paired *t*-test and Wilcoxon signed-rank test. ABC scores were significantly higher than CARS scores. This difference is expected given the distinct scoring ranges and constructs of the two instruments, CARS is a clinician-rated observational measure with a maximum score of 60, while ABC is a parent-reported behavioral checklist with a broader score range. CARS and ABC scores showed a significant moderate positive correlation. This indicates that while the two instruments are related, both capturing aspects of ASD severity, they are not redundant and assess partially distinct dimensions of symptomatology.

In this study, ROC analysis of combined serum biomarkers showed that the panels of NSE + IL-2R + TNF-α, NSE + IL-2R, NSE + TNF-α, and IL-2R + TNF-α all exhibited significant diagnostic value. This highlights the association between serum cytokines (TNF-α, IL-2R) and NSE as a dual-dimensional profiling tool linked to autism symptoms and subtypes, suggesting a potential approach for the biological characterization of ASD, although further studies with dedicated subgroup analyses are needed to confirm this possibility.

Due to the majority of samples with IL-1β, IL-6, and IL-10 falling below the lower detection limit, formal statistical comparisons were not feasible for these cytokines. Therefore, our study does not provide reliable evidence regarding the role of the anti-inflammatory cytokine IL-10 or the pro-inflammatory cytokines (e.g., IL-1β, IL-6) in ASD. Although previous studies have reported associations between these cytokines and immune dysregulation in ASD (Zhao et al., 2021), our findings underscore the need for more sensitive analytical approaches in future research.

## 5 Conclusions

The cytokine alterations observed in this study may contribute to the pathogenesis of autism spectrum disorder (ASD) by mediating an imbalance in pro-inflammatory cytokine expression. Cytokines may not only play important roles in the pathophysiology of ASD but could also offer promise as biomarkers, aiding in earlier diagnosis and therapeutic intervention. Although current research supports an association between immune factors and ASD, more in-depth mechanistic studies are needed to move beyond correlation and clarify precisely how immune dysregulation affects neurodevelopmental trajectories. Therefore, future research should promote systematic molecular and functional experiments to establish the specific mechanistic role of immune factors in ASD-related phenotypes.

Integrating the specific findings on neuron-specific enolase (NSE) and cytokines, our results suggest potential interactions between neuronal injury (reflected by NSE) and immune dysregulation (reflected by cytokine profiles). This interplay may create a vicious cycle in which neuroinflammation and oxidative stress contribute to neuronal damage, which could in turn exacerbate immune abnormalities. The combined assessment of NSE and specific cytokines (such as IL-2R and TNF-α) shows promise for developing a dual-dimensional stratification tool that reflects both neurological and immunological aspects of ASD heterogeneity. This integrative approach offers a new perspective for the biological subtyping of ASD. Future longitudinal studies are essential to validate the dynamic changes in these biomarkers and explore their potential as indicators of treatment response, which may guide the development of targeted interventions addressing these interconnected pathways.

This study has several limitations. The sample size was relatively small, with 41 individuals with ASD and 24 typically developing controls. Accordingly, the current findings should be considered preliminary and warrant replication in larger cohorts. Nevertheless, we would like to highlight that this study was designed as a foundational model for a planned multicenter initiative. The standardized data collection framework developed here is intended to support seamless data aggregation across participating sites in the future. Accumulation of larger samples through this multicenter approach will ultimately allow for more robust subgroup analyses and multivariate modeling, providing more definitive answers to the research questions addressed in this study.

## Ethics statement

This study was approved by the Ethics Committee of the First Affiliated Hospital of Anhui Medical University (No. PJ20230232) and was conducted in accordance with the Declaration of Helsinki. Written informed consent was obtained from the parents or legal guardians of all participants prior to their inclusion in the study.

## Informed consent

Informed consent was obtained from all individual participants included in the study.

## Funding

This work was supported by the 10.13039/501100001809National Natural Science Foundation of China (Grant No. 81472167).

## Conflicts of Interest

The authors have no relevant financial or non-financial interests to disclose.

The authors declare that they have no conflict of interest.
